# Leaky scanning translation generates a second A49 protein that contributes to vaccinia virus virulence

**DOI:** 10.1099/jgv.0.001386

**Published:** 2020-02-25

**Authors:** Sarah Neidel, Alice A. Torres, Hongwei Ren, Geoffrey L. Smith

**Affiliations:** ^1^​ Department of Pathology, University of Cambridge, Tennis Court Road, Cambridge CB2 1QP, UK; ^†^​ Present address: Department of Immunology and Inflammation, Imperial College London, Hammersmith Campus, Du Cane Road, London W12 0NN, UK

**Keywords:** vaccinia virus, gene *A49R*, leaky scanning, NF-κB inhibitor, virulence, multiple functions, Bcl2-fold

## Abstract

Vaccinia virus (VACV) strain Western Reserve gene *A49L* encodes a small intracellular protein with a Bcl-2 fold that is expressed early during infection and has multiple functions. A49 co-precipitates with the E3 ubiquitin ligase β-TrCP and thereby prevents ubiquitylation and proteasomal degradation of IκBα, and consequently blocks activation of NF-κB. In a similar way, A49 stabilizes β-catenin, leading to activation of the wnt signalling pathway. However, a VACV strain expressing a mutant A49 that neither co-precipitates with β-TrCP nor inhibits NF-κB activation, is more virulent than a virus lacking A49, indicating that A49 has another function that also contributes to virulence. Here we demonstrate that gene *A49L* encodes a second, smaller polypeptide that is expressed via leaky scanning translation from methionine 20 and is unable to block NF-κB activation. Viruses engineered to express either only the large protein or only the small A49 protein both have lower virulence than wild-type virus and greater virulence than an *A49L* deletion mutant. This demonstrates that the small protein contributes to virulence by an unknown mechanism that is independent of NF-κB inhibition. Despite having a large genome with about 200 genes, this study illustrates how VACV makes efficient use of its coding potential and from gene *A49L* expresses a protein with multiple functions and multiple proteins with different functions.

## Introduction

Vaccinia virus (VACV) has a large dsDNA genome with approximately 200 genes [1], replicates in the cell cytoplasm [2] and encodes scores of proteins that inhibit the innate response to infection [3]. The genes encoding these immunomodulatory proteins are usually non-essential for virus replication in cell culture, but affect virus virulence and immunogenicity *in vivo*. This paper concerns one of these non-essential genes called *A49L* that is encoded towards the right end of the virus genome.

A49 is a small intracellular protein that is expressed early during infection and is non-essential for virus replication but promotes virus virulence [4]. Crystallography revealed that A49 has a Bcl-2-like fold, and was an unexpected addition to the family of VACV Bcl-2 proteins [5]. Several cellular anti-apoptotic Bcl-2 proteins have a surface groove that binds the BH3 peptide of pro-apoptotic Bcl-2 proteins and thereby inhibit their pro-apoptotic activity. However, A49 lacks this surface groove and, consistent with this, did not bind pro-apoptotic Bcl-2 proteins Bax and Bak [5], which are bound by the closest orthologue of A49, the myxoma virus protein M11 [6].

The first function assigned to A49 was the inhibition of NF-κB activation by molecular mimicry [4]. Near the N terminus of A49 there is a short amino acid sequence containing two serines (S7 and S12) that are conserved in several cellular proteins, such as IκBα and β-catenin, and also some virus proteins, such as HIV-1 Vpu. In IκBα these serines can be phosphorylated, leading to the recognition of p-IκBα by the E3 ligase β-TrCP, and its consequential ubiquitylation and proteosomal degradation. In turn, this enables release of the NF-κB subunits into the nucleus and transcription of NF-κB-responsive genes. A49 co-precipitates with β-TrCP and this interaction was abrogated by mutation of S7 and S12 to alanine [4]. By interacting with β-TrCP, A49 prevents β-TrCP-mediated ubiquitylation of p-IκBα, and so IκBα remains bound to the NF-κB subunits in the cytoplasm. Another substrate of β-TrCP is β-catenin, which was also shown to be stabilized by A49, leading to its accumulation in cells and the consequential activation of the wnt signalling pathway [7].

Recently, further insight to the mechanism of action of A49 showed that phosphorylation of S7 was necessary for A49 to bind β-TrCP and inhibit activation of NF-κB, whereas S12 was dispensable for these functions [8]. Therefore, A49 is a conditional inhibitor of NF-κB and requires activation by phosphorylation to become an inhibitor of this pathway. A kinase that phosphorylates A49 S7 was identified as IKKβ, the same kinase that phosphorylates IκBα and leads to activation of the NF-κB pathway. Consequently, A49 is activated to become an inhibitor of NF-κB when the pathway leading to NF-κB activation is switched on. In other words, NF-κB activation is a turn on for A49 to turn off NF-κB activation [8]. This report also showed that A49 has a second function that contributes to virulence, independent of NF-κB inhibition. VACV strains expressing a mutant A49 protein that either cannot bind β-TrCP and so cannot inhibit NF-κB activation, or does these functions constitutively, both had virulence that was greater than that of vΔA49 and lower than that of wild-type (WT) virus [8].

Here another unexpected feature of the *A49L* gene is described. The data presented show that *A49L* encodes two polypeptides that are translated by initiation from either methionine 1 (M1) or methionine 20 (M20) with the same temporal kinetics. The smaller protein lacking the first 19 aa does not bind β-TrCP and so cannot inhibit NF-κB activation. Nonetheless, viruses engineered to express only the large or small protein each had reduced virulence compared to WT virus and greater virulence than a deletion mutant lacking both proteins. This showed that the small protein has a function that contributes to virulence and that is not provided by the large protein. This study illustrates how VACV makes very efficient use of its coding potential.

## Methods

### Cells and viruses

HEK-293T, CV-1 and BSC-1 cells were grown in Dulbecco’s modified Eagle’s medium (DMEM; Gibco) supplemented with heat-treated (56 °C, 1 h) 10 % foetal bovine serum (FBS; Harlan-Sera Lab), 100 U ml^−1^ penicillin and 100 µg ml^−1^ streptomycin (P and S). RK_13_ cells were grown in minimum essential medium (MEM) containing 10 % FBS and P and S. VACV strain Western Reserve (WR) and mutants thereof were used. A plaque-purified WT virus (vA49R) and a deletion mutant lacking the *A49L* gene (vΔA49L) were described [4]. Additional VACVs bearing mutant *A49L* alleles were constructed by transient dominant selection [9], starting with vΔA49L as described by Neidel *et al*. [8]. Briefly, cells were infected with vΔA49L and then transfected with a plasmid containing the mutant *A49L* gene and flanking sequences. Recombinant viruses containing the transfected plasmid were selected by plaque assay on BSC-1 cells in the presence of mycophenolic acid, xanthine and hypoxanthine. These intermediate viruses were then resolved to the final recombinant virus by plaque assay in the absence of drugs and screened for the *A49L* gene by polymerase chain reaction (PCR). The *A49L* gene locus was sequenced for all viruses to confirm the genotype. Stocks of VACV were grown on RK_13_ cells and titrated by plaque assay on BSC-1 cells.

### Antibodies and cytokines

Tumour necrosis factor alpha (TNFα) and interleukin-1β (IL-1β) were bought from Peprotech. The following antibodies were used, each diluted 1 : 1 000: rabbit polyclonal against VACV protein A49 [4], mouse monoclonal AB1.1 against VACV protein D8 [10]. Mouse monoclonal against anti-α-tubulin (DM1A; Millipore) was used diluted 1 : 10 000.

### Reporter gene assay

The activation of the NF-κB pathway was measured by reporter gene assay as described by Mansur *et al*. [4]. Briefly, a reporter plasmid bearing an NF-κB-responsive promoter linked to firefly luciferase was transfected into HEK-293T cells together with either empty vector (EV) or the same plasmid expressing A49 or mutants thereof. Cells were also transfected with a plasmid expressing *Renilla* luciferase as a transfection control. The following day cells were stimulated with 15 ng ml^−1^ TNFα for 8.5 h or IL-1β for 8 h (as indicated). Then, cell lysates were prepared and firefly and *Renilla* luciferase activities were measured. Data are expressed as the firefly activity normalized to the *Renilla* activity and the fold increase relative to unstimulated control. Three replicates were included for each condition and all experiments were conducted three times. Data from a representative experiment are shown. Statistical analysis compared the fold increase in the presence of EV to individual A49 mutants.

### Mutagenesis

The *A49L* open reading frame was mutated using the QuickChange Site-Directed Mutagenesis Kit (Agilent) and the fidelity of all mutants was confirmed by DNA sequencing.

### Polyacrylamide gel electrophoresis and immunoblotting

Extracts of infected or transfected cells were prepared and analysed by SDS-polyacrylamide gel electrophoresis (SDS-PAGE) and immunoblotting with the indicated antibodies as described by Mansur *et al*. [4]. The positions of molecular mass markers in kDa are shown on the left of each immunoblot.

### *In vivo* infections

The virulence of the indicated VACVs compared to WT VACV WR was measured in a murine intranasal model as described by Alcami and Smith [11]. Groups (*n*=5) of female BALB/c mice (6–8 weeks old) were infected intranasally (in both nostrils) under anaesthetic with 5×10^3^ plaque-forming units (p.f.u.) of the indicated virus. The titre of the diluted virus used for infection was determined by plaque assay to confirm the dose administered. The body weight of each mouse was recorded daily and compared to its weight on day zero. Data for each group are expressed as the sem. Each experiment was conducted twice and the data shown are representative.

### Statistical analysis

Data were analysed by unpaired Student’s *t*-test or a two-way analysis of variance (ANOVA) test with GraphPad Prism statistical software (GraphPad Software). Statistical significance is expressed as: *P<0.05, **P<0.01, ***P<0.001.

## Results

### The *A49L* gene encodes a second polypeptide

**Fig. 1.**
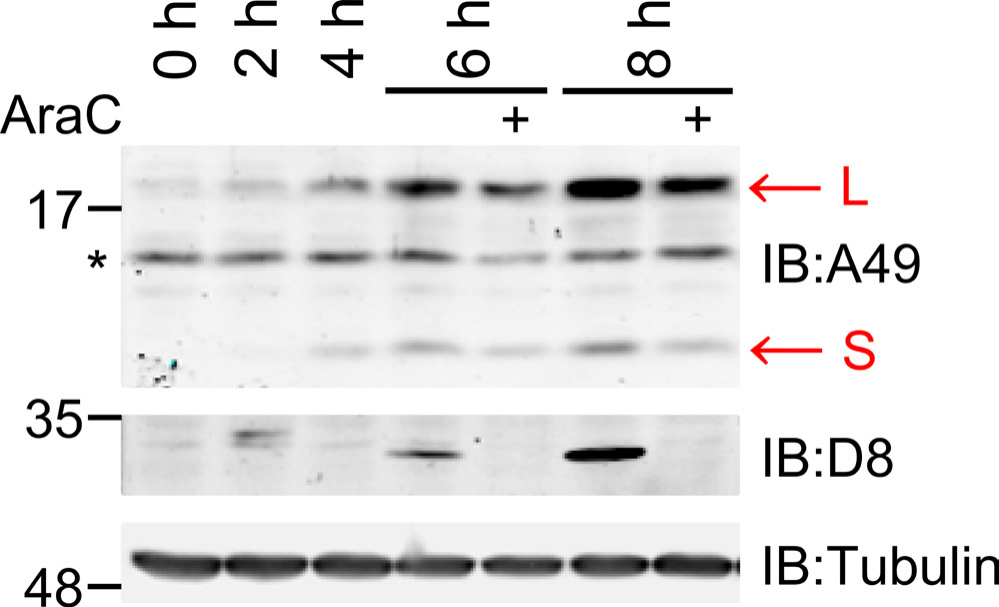
Two proteins are encoded from the *A49L* gene. HEK-293T cells were infected with VACV WR at 5 p.f.u./cell. Where indicated, 40 µg ml^−1^ cytosine arabinoside (AraC) was added. At 2, 4, 6 or 8 h p.i. cells were harvested and cell extracts were analysed by SDS-PAGE and immunoblotting (IB) with antibodies to the VACV proteins A49 and D8 and cellular α-tubulin. Red arrows mark the positions of the large (L) and small (S) A49 proteins and the star marks a nonspecific signal also present at 0 h.

A rabbit polyclonal antibody raised against the A49 protein [4] was used to analyse A49 proteins in VACV-infected cells at different times post-infection (p.i.) (Fig. 1). Immunoblotting showed that an A49 protein of ~18 kDa (L) was detected by 2–4 h p.i. This protein was present in the presence of cytosine arabinoside (AraC), and so was expressed prior to virus DNA replication. The reduced levels in the presence of AraC also indicated expression late during infection. In addition, a smaller and less abundant protein of ~11 kDa (S) was also detected with the same antibody and appeared with the same kinetics. Immunoblotting for VACV structural protein D8 with a mouse monoclonal antibody [10] confirmed the effectiveness of AraC in blocking late virus gene expression, and immunoblotting for α-tubulin confirmed equal loading of samples. Analysis of the *A49L* open reading frame revealed a second AUG at codon 20 that might be used to make the smaller protein.

### The smaller A49 protein is made by leaky scanning translation

**Fig. 2.**
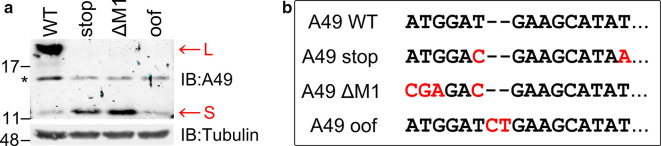
The A49 S protein is expressed by leaky scanning translation. (a) Immunoblot. HEK-293T cells were transfected with plasmids encoding WT A49 or mutants [as shown in (b)]. The following day cell extracts were prepared and analysed by SDS-PAGE and immunoblotting with antibodies against A49 (top) and α-tubulin (bottom). Red arrows mark the large (L) and small (S) A49 proteins. (b) Nucleotide sequence of the WT and mutant A49 alleles used in (a) starting from the ATG codon 1 of the full-length open reading frame. Positions mutated from the WT sequence are shown in red. A49 stop contains an in-frame termination codon at nucleotides 13–15 and a T to C substitution at nucleotide 6 to remove an OOF ATG codon. A49 ΔM1 has the ATG codon (positions 1–3) changed to CGA, and the same T to C change at position 6 as for A49 stop. A49 OOF has an insertion of two nucleotides after nucleotide 6 to put the ATG codons at codon 1 and 20 in different reading frames.

To address the nature of the smaller protein and determine how it was expressed, the *A49L* reading frame was mutated (nucleotide 15 T to A) to introduce a termination codon between M1 and M20 (called stop). An existing out-of-frame ATG codon (nucleotides 5–8) was also mutated (ATG to ACG) to prevent any ribosomal initiation from this position. A second mutant with the same ATG to ACG change and in which the first ATG codon was mutated to CGA (ΔM1) was also constructed. Plasmids encoding WT and mutant *A49L* genes were transfected into HEK-293T cells and protein extracts were analysed by immunoblotting (Fig. 2a). This showed that the introduction of a termination codon between the first and second in-frame AUG, or the removal of the first AUG, both prevented expression of the full-length A49 protein and enhanced the expression of the smaller protein. This suggested that after binding to the 5′ cap structure of A49 mRNA, ribosomes scan along the mRNA and initiate translation from the first available AUG codon. A third mutant in which two additional nucleotides were introduced between M1 and M20 (A49 out of frame – OOF) was also constructed and analysed (Fig. 2b). This allele failed to express the full-length protein, as expected, due to a switch of reading frame after codon 2 and the presence of termination codons in the +2 reading frame shortly downstream. Note that the A49 antibody would also fail to detect any protein made by such a short polypeptide from this different reading frame. However, consistent with leaky scanning, this allele expressed the smaller protein.

### Mutation of methionine 20 compromises A49 function

**Fig. 3.**
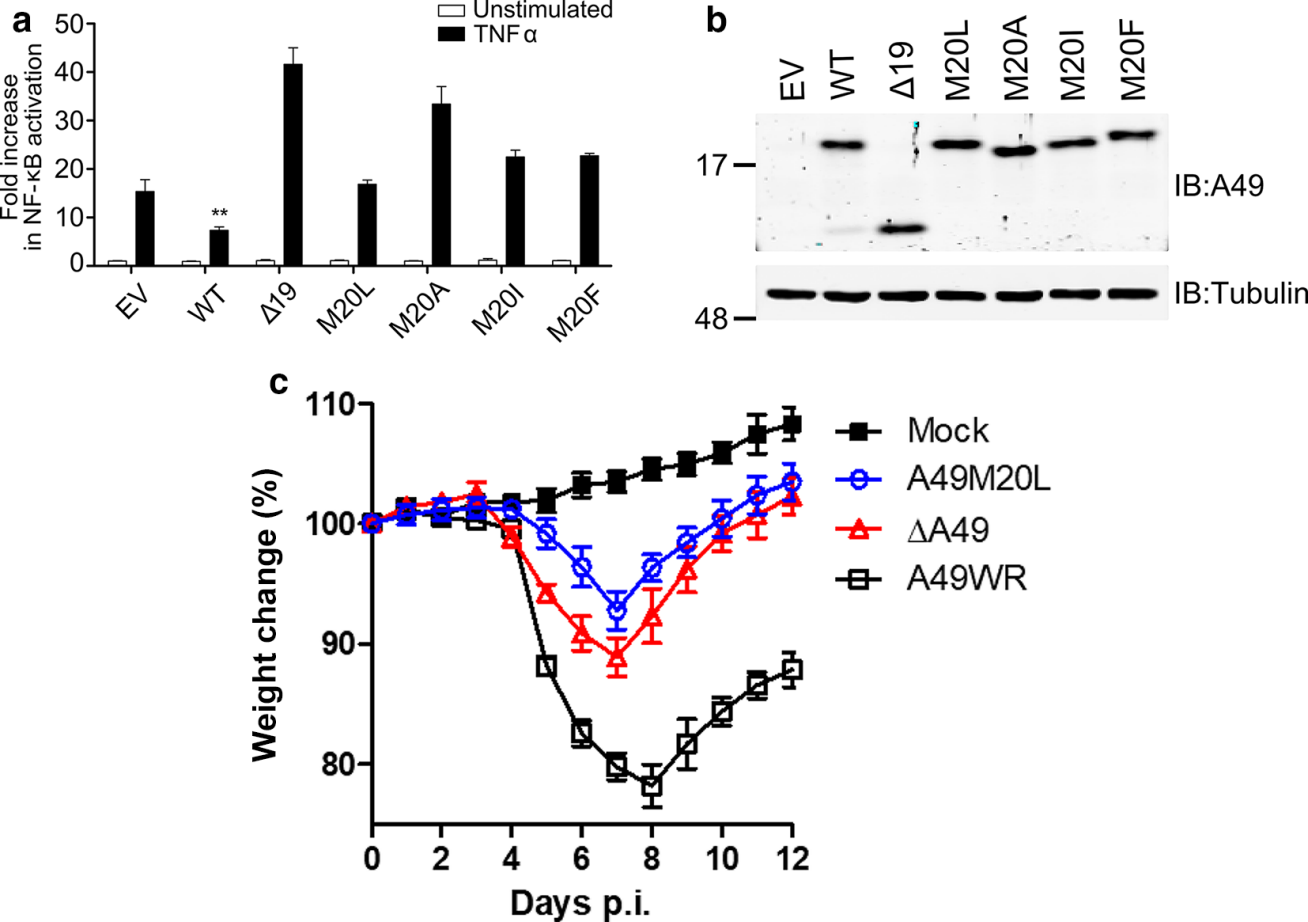
Mutation of methionine 20 abrogates A49 L function. (a) Reporter gene assay. HEK-293T cells were transfected with empty vector (EV), or plasmids expressing WT A49 or mutant A49 proteins either lacking the N-terminal 19 aa (Δ19) or with M20 changed to L, A, I or F (M20L, M20A, M20I and M20F). The following day, cells were stimulated (black bars) with TNF-α (15 ng ml^−1^ for 8.5 h) and then lysates were prepared and the *Renilla* and firefly luciferase activity were measured. Data presented are the mean of the firefly luciferase activity normalized to the *Renilla* luciferase activity (each in triplicate), which were then normalized to unstimulated EV±sd. **P<0.01 compared to stimulated EV. (b). Immunoblot. Cell extracts, prepared as in (a), were analysed by SDS-PAGE and immunoblotting with antibodies against VACV protein A49 (top) or α-tubulin (bottom). (c) Virulence measurement of VACVs. Groups (*n*=5) of female BALB/c mice were infected intranasally with 5×10^3^ p.f.u. of indicated VACVs or were mock-infected (mock) and the weight of each mouse was measured daily. The weight of each mouse was compared to its weight on day zero and the data are presented as the mean±sem for each group. Statistical analysis was by two-way ANOVA test. The data from A49M20L and vΔA49 were each significantly different from A49WR (*P*=0.0001 WR versus vΔA49, and *P*=0.001 WR versus vM20L), but were not significantly different from each other.

The mutants A49 stop and A49 ΔM1 each made only the small A49 protein, as did another mutant lacking the first 19 codons (Δ19). To make an allele that made only the larger protein, the M20 codon was mutated to leucine, alanine, isoleucine or phenylalanine. Transfection of these alleles into HEK-293T cells gave expression of all proteins at similar levels, albeit with slightly different electrophoretic mobility (Fig. 3b). Next, the ability to these alleles to inhibit NF-κB activation was measured by reporter gene assay (Fig. 3a). Surprisingly, all the mutants with M20 changed to another amino acid lacked the ability to inhibit NF-κB activation, despite retaining the upstream residues (including S7) needed for interaction with β-TrCP. The Δ19 mutant was also unable to inhibit the pathway, as expected (Fig. 3a). The virulence of the A49 M20L protein was analysed *in vivo* after insertion of this mutant allele into the VACV lacking the *A49L* gene (vΔA49) [4]. As observed previously [4, 8], the vΔA49 mutant was attenuated compared to the WT virus, as shown by reduced weight loss after intranasal infection of Balb/c mice. Surprisingly, the virulence of the A49 M20L mutant virus was equivalent to that of its parent virus, vΔA49, indicating that the larger A49 protein with M20L did not contribute to virulence and seemed non-functional. This result prevented a comparison of the contribution of the large and small A49 proteins to virulence and so additional mutants were designed.

### A mutant making only the large A49 protein

**Fig. 4.**
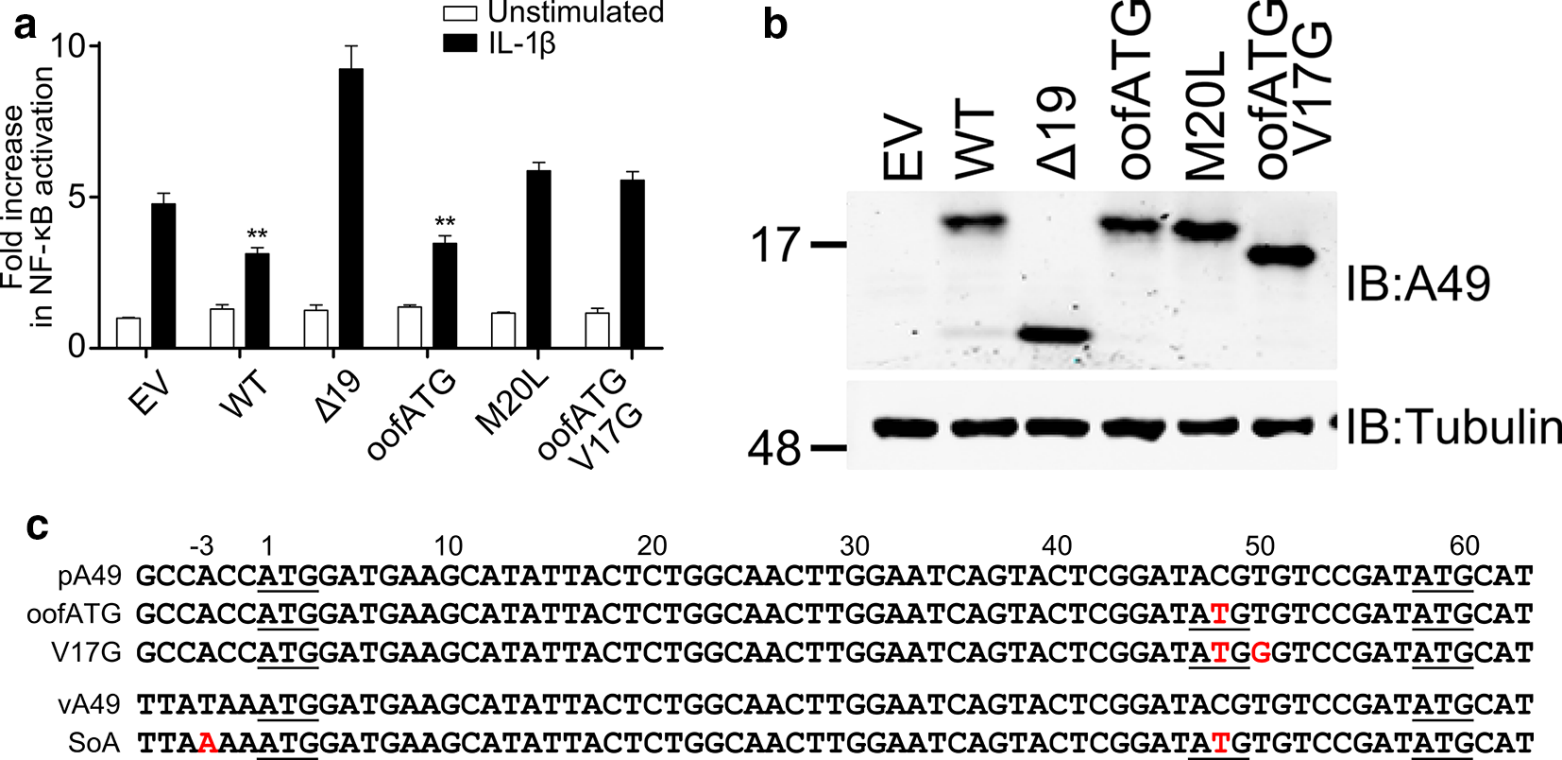
Analysis of A49 alleles making either only the large or small protein. (a) Reporter gene assay using plasmids described in (c) and in Figs 2 and 3, and conducted as described in Fig. 3. (b) Immunoblot (IB) showing expression of the different A49 proteins using extracts from cells prepared as in (a). (c) Nucleotide sequence of WT A49 and mutants. Changes from the WT sequence are shown in red. The top three rows are sequences present in plasmids expressing these A49 alleles and the bottom two rows show the sequence of WT or SoA (strong first ATG in oofATG) mutant virus. oofATG (out-of-frame ATG) is mutated to create an ATG codon at nucleotides 47–49, between the ATG codons at positions 1 and 20. V17G is like oofATG but also has nucleotide 50 changed from T to G to give the new ATG codon a stronger Kozak consensus sequence. SoA has the T at position −3 changed to A to give the codon 1 AUG a stronger Kozak consensus sequence.

All A49 mutants with M20 altered to another amino acid were unable to inhibit NF-κB activation and A49 M20L made no contribution to virulence *in vivo*. Therefore, an alternative mutant that made only the larger protein was designed. To prevent expression of the smaller protein an additional ATG codon was created between codon 1 and 20 that was out of frame with the A49 reading frame. Ribosomes failing to initiate translation at codon 1 would now scan the mRNA and initiate translation from the new AUG codon (nucleotides 47–49) rather than codon 20. This mutant is called out-of-frame ATG (OOF ATG). This new initiation codon has a sub-optimal Kozak consensus sequence, and this was strengthened by substitution of nucleotide 50 from T to G. This change also caused a conservative amino acid substitution in the large protein (V17G), so this mutant was called oofATG V17G (Fig. 4c). The expression and function of these mutant proteins was tested by transfection of plasmids containing these alleles followed by immunoblotting, and by reporter gene assay (Fig. 4a, b). This showed that oofATG and oofATG V17G each expressed only the larger protein and at levels similar to WT. The reporter gene assay showed that oofATG inhibited activation of NF-κB, whereas oofATG V17G and M20L did not.

**Fig. 5.**
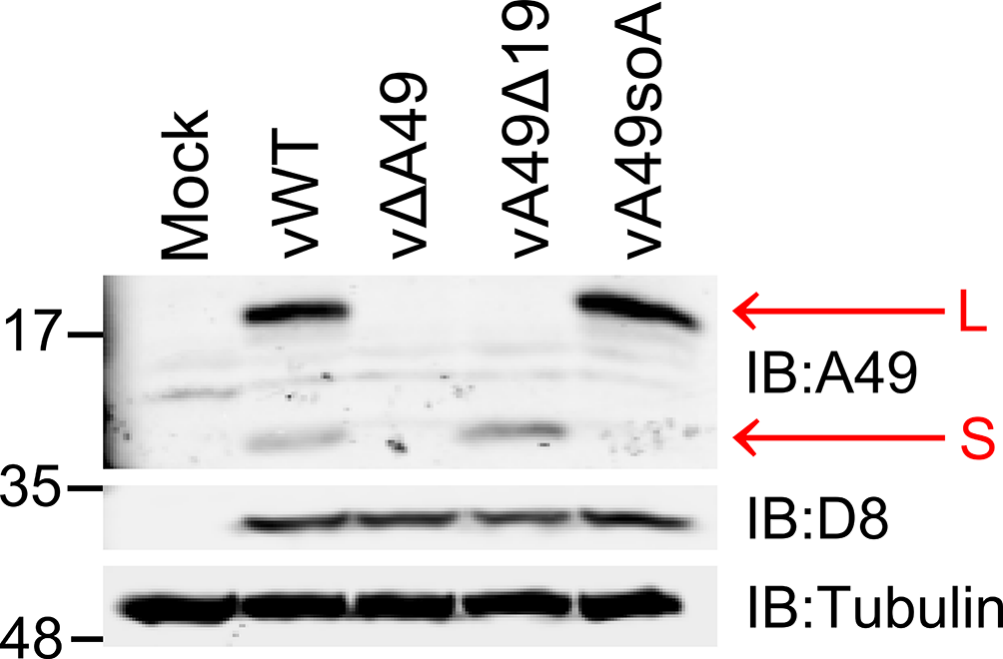
Expression of A49 proteins in cells infected with VACVs. HEK-293T cells were infected at 5 p.f.u./cell with WT VACV (vWT) or viruses designed to make neither A49 protein (vΔA49), only the large (L) protein (vA49SoA), or only the small protein vA49Δ19. At 16 h p.i. cells were harvested and cell extracts were analysed by SDS-PAGE and immunoblotting with antibodies to VACV proteins A49 and D8 or α-tubulin. Red arrows indicate the positions of the large (L) or small (S) A49 proteins.

Expression of these mutant A49 alleles from plasmid vectors showed that oofATG and oofATG V17G each expressed only the full-length protein. However, when the oofATG allele was introduced into the vΔA49 VACV and the expression of A49 was examined, a low level of expression of the small A49 protein remained (Fig. S1, available in the online version of this article). The only difference between the sequences of the oofATG allele in the plasmid and the virus was the presence of a T at the −3 position in the virus and an A at the −3 position in the plasmid. Since the presence of an A at −3 strengthens the Kozak consensus sequence, a virus (SoA) was engineered to have A rather than T at nucleotide −3. Analysis of the expression of the A49 proteins from cells infected by this virus showed that only the large protein was present (Fig. 5). A trace band at the same position as the small protein was detected, but this was also seen in cells infected with the A49 deletion mutant and therefore represents a background cross-reacting band.

### Both the large and small A49 proteins contribute to virulence

**Fig. 6.**
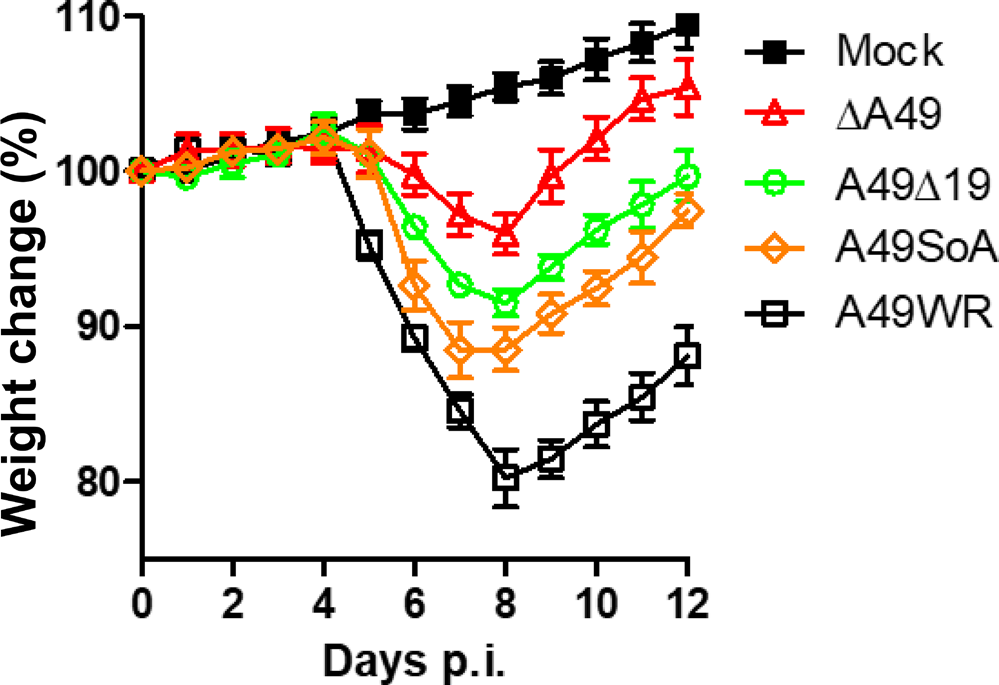
Virulence of VACVs. Groups of mice were infected with the indicated viruses as in Fig. 3c and were weighed daily. Data were analysed and are expressed as described in Fig. 3c. Statistical analysis was by two-way ANOVA test. Groups vA49Δ19 and vA49SoA were not significantly different from each other, but were each significantly different from both vΔA49 and A49WR (*P*=0.01 soA vs WR and soA versus vΔA49).

Now that viruses expressing only the large A49 protein (SoA), only the small A49 protein (A49Δ19), neither protein (vΔA49), or both proteins (WT) were available, the contributions of the two proteins to virulence was examined *in vivo* (Fig. 6). This showed that the virulence of viruses expressing only the small protein, or only the large protein, was greater than that of the deletion mutant and lower than that of the WT virus, and therefore both proteins contribute to virulence. Since the smaller protein cannot inhibit NF-κB, it must contribute to virulence by an independent mechanism. Similarly, the large protein alone is a virulence factor but lacks something that the smaller protein provides. This might be explained by the N-terminal 19 aa masking some part of A49 that would otherwise be exposed and have a function that promotes virulence.

## Discussion

The VACV genome encodes approximately 200 genes and so is large in comparison with many DNA and all RNA viruses. Yet, despite this, the virus utilizes its coding capacity efficiently by having tightly packed genes, no introns and many multi-functional proteins. The *A49L* gene of VACV WR is a good example of this efficient use of coding capacity. Previously, the A49 protein was shown to inhibit the activation of NF-κB and promote activation of the wnt signalling pathway by engaging the E3 ubiquitin ligase β-TrCP [4, 7]. Interaction with β-TrCP stabilizes substrates of β-TrCP such as IκBα and β-catenin that otherwise would be ubiquitylated and degraded. However, this is not the only function of A49. Evidence for an additional function came from analysis of a virus expressing a mutant A49 protein that can neither engage β-TrCP nor block NF-κB activation. The virulence of this virus was greater than that of vΔA49 but lower than that of WT virus [8], indicating that A49 has another function. Consistent with this, a virus expressing A49 that binds β-TrCP constitutively has reduced virulence compared to WT virus, suggesting that being free to interact with other substrates might also be important for virulence [8].

Here, an additional feature of the *A49L* gene is described. In addition to encoding a full-length protein of 162 amino acids, the *A49L* gene also expresses a smaller protein that is expressed by translational initiation from M20 of the larger protein. This smaller protein is less abundant than the full-length A49 protein, but is expressed with the same temporal kinetics. Removal of the N-terminal 19 aa, mutation of the first AUG codon, or creation of a stop codon between the AUG codons at position 1 and 20, all resulted in expression of only the smaller protein that is unable to inhibit NF-κB activation because it lacks the conserved motif SGXX(X)S (amino acids 7–12 in A49) that is present in β-TrCP substrates. Nonetheless, a mutant virus expressing only the smaller A49 protein had greater virulence than the A49 deletion mutant, showing that the smaller protein has another function that contributes to virulence independent of NF-κB inhibition.

Attempts to engineer a virus that expressed only the large A49 protein (and which was functional) were thwarted by the unexpected finding that mutation of M20 to either A, F, I or L resulted in an A49 protein that was unable to inhibit NF-κB and, in the case of M20L, did not contribute to virulence (the other mutants were not tested). Further mutations in this short N-terminal region, such as V17G (Fig. 4) or L10V (unpublished data), also resulted in loss of inhibition of NF-κB activation. Therefore, an alternative strategy to prevent expression of the small A49 protein was used. An additional (out-of-frame) AUG codon was introduced between codon 1 and codon 20, preventing access of ribosomes to the codon 20 AUG. This, together with mutation of the −3 position of codon 1 to strengthen the Kozak consensus sequence [12], resulted in a virus that only made the large A49 protein of WT sequence. In a murine intranasal model, this virus had greater virulence than the deletion mutant, but lower virulence than WT virus. Therefore, although the large A49 protein includes the entire sequence of the smaller protein, it cannot undertake some function of the small protein that contributes to virulence. This function is unknown but is independent of β-TrCP binding and modulation of the NF-κB and wnt signalling pathways.

Collectively, these data show that the *A49L* gene encodes a full-length protein with multiple functions, and multiple proteins with different functions. This remarkably efficient use of coding capacity might be considered unusual for a large DNA virus, but may not turn out to be so as the functions of the individual genes and proteins of these viruses are studied in greater depth. As mentioned in the introduction, the A49 protein is a member of the VACV family of Bcl-2 proteins [5]. There are 11 members of this family and the majority of these small alpha helical proteins have multiple functions in immune evasion. Examples include: the N1 protein that has anti-apoptotic and NF-κB inhibitory activity [13–15]; the F1 protein that inhibits apoptosis and activation of the inflammasome [16–18]; the B14 protein that inhibits NF-κB activation [19] but also has another function that contributes to virulence (unpublished data); the K7 [20–22] and A46 [23–27] proteins that each inhibit activation of both the IRF-3 and NF-κB pathways; and the C6 protein that inhibits activation of both the IRF-3 pathway [28] and the JAK-STAT pathway downstream of type I interferon binding to its receptor [29], and induces proteolytic degradation of histone deacetylases 4 and 5 [30, 31].

Outside of the Bcl-2 family, other VACV proteins engaged in immune evasion also have multiple functions. Examples include protein E3 that binds dsRNA via its C-terminal domain and prevents dsRNA-dependent activation of protein kinase R [32] and 2−5′-oligoadenylate synthetase [33]. The N-terminal region has a Z-DNA-binding domain [34] and via this domain inhibits DAI-dependent necroptosis [35]. Both domains contribute to virus virulence [36]. Other multifunctional VACV proteins are the related proteins C16 and C4. Protein C16 inhibits Ku-mediated DNA sensing via its C-terminal domain [37] and induces a hypoxic response by binding the oxygen sensor PHD2 via its N-terminal domain [38], leading to reprogramming of cellular energy metabolism [39]. Protein C4 inhibits NF-κB activation [40] and the Ku-mediated DNA sensing [41]. Protein A55 is another multifunctional, multi-domain protein. A55 binds cullin 3 via its N-terminal BTB domain [42, 43], and inhibits NF-κB by binding to the importin KPNA2 to prevent p65 translocation into the nucleus [43]. Another multifunctional orthopoxvirus protein, which is secreted from the cell, is the protein G2 of variola virus (VARV), the cause of smallpox. This protein, and orthologues in monkeypox virus and cowpox virus, binds TNF via its N-terminal cysteine-rich domain and chemokines via a C-terminal domain [44].

While many proteins are multi-functional, few VACV genes encode multiple proteins. One example is the *E3L* gene that, like A49, encodes two different proteins that differ at their N-termini due to translational initiation from different in-frame AUG codons [45]. Both the 25 and 19 kDa E3 proteins contain the C-terminal dsRNA-binding domain, but a function for the smaller protein not performed by the larger protein has not been described, and its biological importance is unknown. Other VACV genes encode proteins that are cleaved proteolytically, for instance during capsid maturation, to give distinct polypeptides. In addition to the proteins predicted to be encoded by the VACV genome from analysis of the genome sequence [1], ribosomal profiling provided evidence for translation of additional VACV polypeptides [46]. Interestingly, the shorter polypeptides encoded by the *E3L* and *A49L* genes were not detected by this technology.

In summary, the VACV *A49L* gene is shown here to encode two different proteins that are expressed by translational initiation from different in-frame AUG codons. Both proteins contribute to virus virulence and although the sequence of the smaller protein is present entirely within the larger protein, it has an unknown function that the larger protein does not provide.

## Supplementary Data

Supplementary material 1

## References

[1] Goebel SJ, Johnson GP, Perkus ME, Davis SW, Winslow JP (1990). The complete DNA sequence of vaccinia virus. Virology.

[2] Moss B, Knipe DMH (2013). Poxviridae. Fields Virology.

[3] Smith GL, Benfield CTO, Maluquer de Motes C, Mazzon M, Ember SWJ (2013). Vaccinia virus immune evasion: mechanisms, virulence and immunogenicity. J Gen Virol.

[4] Mansur DS, Maluquer de Motes C, Unterholzner L, Sumner RP, Ferguson BJ (2013). Poxvirus targeting of E3 ligase β-TrCP by molecular mimicry: a mechanism to inhibit NF-κB activation and promote immune evasion and virulence. PLoS Pathog.

[5] Neidel S, Maluquer de Motes C, Mansur DS, Strnadova P, Smith GL (2015). Vaccinia virus protein A49 is an unexpected member of the B-cell Lymphoma (Bcl)-2 protein family. J Biol Chem.

[6] Kvansakul M, van Delft MF, Lee EF, Gulbis JM, Fairlie WD (2007). A structural viral mimic of prosurvival Bcl-2: a pivotal role for sequestering proapoptotic bax and bak. Mol Cell.

[7] Maluquer de Motes C, Smith GL (2017). Vaccinia virus protein A49 activates Wnt signalling by targetting the E3 ligase β-TrCP. J Gen Virol.

[8] Neidel S, Ren H, Torres AA, Smith GL (2019). NF-κB activation is a turn on for vaccinia virus phosphoprotein A49 to turn off NF-κB activation. Proc Natl Acad Sci U S A.

[9] Falkner FG, Moss B (1990). Transient dominant selection of recombinant vaccinia viruses. J Virol.

[10] Parkinson JE, Smith GL (1994). Vaccinia virus gene A36R encodes a Mr 43-50 K protein on the surface of extracellular enveloped virus. Virology.

[11] Alcami A, Smith GL (1992). A soluble receptor for interleukin-1β encoded by vaccinia virus: a novel mechanism of virus modulation of the host response to infection. Cell.

[12] Kozak M (1986). Point mutations define a sequence flanking the AUG initiator codon that modulates translation by eukaryotic ribosomes. Cell.

[13] Cooray S, Bahar MW, Abrescia NGA, McVey CE, Bartlett NW (2007). Functional and structural studies of the vaccinia virus virulence factor N1 reveal a Bcl-2-like anti-apoptotic protein. J Gen Virol.

[14] Graham SC, Bahar MW, Cooray S, Chen RA-J, Whalen DM (2008). Vaccinia virus proteins A52 and B14 share a Bcl-2-like fold but have evolved to inhibit NF-κB rather than apoptosis. PLoS Pathog.

[15] Maluquer de Motes C, Cooray S, Ren H, Almeida GMF, McGourty K (2011). Inhibition of apoptosis and NF-κB activation by vaccinia protein N1 occur via distinct binding surfaces and make different contributions to virulence. PLoS Pathog.

[16] Kvansakul M, Yang H, Fairlie WD, Czabotar PE, Fischer SF (2008). Vaccinia virus anti-apoptotic F1L is a novel Bcl-2-like domain-swapped dimer that binds a highly selective subset of BH3-containing death ligands. Cell Death Differ.

[17] Wasilenko ST, Banadyga L, Bond D, Barry M (2005). The vaccinia virus F1L protein interacts with the proapoptotic protein bak and inhibits bak activation. J Virol.

[18] Gerlic M, Faustin B, Postigo A, Yu EC-W, Proell M (2013). Vaccinia virus F1L protein promotes virulence by inhibiting inflammasome activation. Proc Natl Acad Sci U S A.

[19] Chen RA-J, Ryzhakov G, Cooray S, Randow F, Smith GL (2008). Inhibition of IκB kinase by vaccinia virus virulence factor B14. PLoS Pathog.

[20] Benfield CTO, Ren H, Lucas SJ, Bahsoun B, Smith GL (2013). Vaccinia virus protein K7 is a virulence factor that alters the acute immune response to infection. J Gen Virol.

[21] Kalverda AP, Thompson GS, Vogel A, Schröder M, Bowie AG (2009). Poxvirus K7 protein adopts a Bcl-2 fold: biochemical mapping of its interactions with human DEAD box RNA helicase DDX3. J Mol Biol.

[22] Schröder M, Baran M, Bowie AG (2008). Viral targeting of dead box protein 3 reveals its role in TBK1/IKKepsilon-mediated IRF activation. Embo J.

[23] Bowie A, Kiss-Toth E, Symons JA, Smith GL, Dower SK (2000). A46R and A52R from vaccinia virus are antagonists of host IL-1 and Toll-like receptor signaling. Proc Natl Acad Sci U S A.

[24] Fedosyuk S, Bezerra GA, Radakovics K, Smith TK, Sammito M (2016). Vaccinia virus immunomodulator A46: a lipid and protein-binding scaffold for sequestering host TIR-domain proteins. PLoS Pathog.

[25] Fedosyuk S, Grishkovskaya I, de Almeida Ribeiro E, Skern T (2014). Characterization and structure of the vaccinia virus NF-κB antagonist A46. J Biol Chem.

[26] Stack J, Bowie AG (2012). Poxviral protein A46 antagonizes Toll-like receptor 4 signaling by targeting BB loop motifs in Toll-IL-1 receptor adaptor proteins to disrupt receptor:adaptor interactions. J Biol Chem.

[27] Stack J, Haga IR, Schröder M, Bartlett NW, Maloney G (2005). Vaccinia virus protein A46R targets multiple toll-like-interleukin-1 receptor adaptors and contributes to virulence. J Exp Med.

[28] Unterholzner L, Sumner RP, Baran M, Ren H, Mansur DS (2011). Vaccinia virus protein C6 is a virulence factor that binds TBK-1 adaptor proteins and inhibits activation of IRF3 and IRF7. PLoS Pathog.

[29] Stuart JH, Sumner RP, Lu Y, Snowden JS, Smith GL (2016). Vaccinia virus protein C6 inhibits type I IFN signalling in the nucleus and binds to the transactivation domain of STAT2. PLoS Pathog.

[30] Lu Y, Stuart JH, Talbot-Cooper C, Agrawal-Singh S, Huntly B (2019). Histone deacetylase 4 promotes type I interferon signaling, restricts DNA viruses, and is degraded via vaccinia virus protein C6. Proc Natl Acad Sci U S A.

[31] Soday L, Lu Y, Albarnaz JD, Davies CTR, Antrobus R (2019). Quantitative temporal proteomic analysis of vaccinia virus infection reveals regulation of histone deacetylases by an interferon antagonist. Cell Rep.

[32] Chang HW, Watson JC, Jacobs BL (1992). The E3L gene of vaccinia virus encodes an inhibitor of the interferon-induced, double-stranded RNA-dependent protein kinase. Proc Natl Acad Sci U S A.

[33] Rivas C, Gil J, Mĕlková Z, Esteban M, Díaz-Guerra M (1998). Vaccinia virus E3L protein is an inhibitor of the interferon (i.f.n.)-induced 2-5A synthetase enzyme. Virology.

[34] Kwon J-A, Rich A (2005). Biological function of the vaccinia virus Z-DNA-binding protein E3L: gene transactivation and antiapoptotic activity in HeLa cells. Proc Natl Acad Sci U S A.

[35] Koehler H, Cotsmire S, Langland J, Kibler KV, Kalman D (2017). Inhibition of DAI-dependent necroptosis by the Z-DNA binding domain of the vaccinia virus innate immune evasion protein, E3. Proc Natl Acad Sci U S A.

[36] Brandt TA, Jacobs BL (2001). Both carboxy- and amino-terminal domains of the vaccinia virus interferon resistance gene, E3L, are required for pathogenesis in a mouse model. J Virol.

[37] Peters NE, Ferguson BJ, Mazzon M, Fahy AS, Krysztofinska E (2013). A mechanism for the inhibition of DNA-PK-mediated DNA sensing by a virus. PLoS Pathog.

[38] Mazzon M, Peters NE, Loenarz C, Krysztofinska EM, Ember SWJ (2013). A mechanism for induction of a hypoxic response by vaccinia virus. Proc Natl Acad Sci U S A.

[39] Mazzon M, Castro C, Roberts LD, Griffin JL, Smith GL (2015). A role for vaccinia virus protein C16 in reprogramming cellular energy metabolism. J Gen Virol.

[40] Ember SWJ, Ren H, Ferguson BJ, Smith GL (2012). Vaccinia virus protein C4 inhibits NF-κB activation and promotes virus virulence. J Gen Virol.

[41] Scutts SR, Ember SW, Ren H, Ye C, Lovejoy CA (2018). Dna-Pk is targeted by multiple vaccinia virus proteins to inhibit DNA sensing. Cell Rep.

[42] Gao C, Pallett MA, Croll TI, Smith GL, Graham SC (2019). Molecular basis of cullin-3 (Cul3) ubiquitin ligase subversion by vaccinia virus protein A55. J Biol Chem.

[43] Pallett MA, Ren H, Zhang R-Y, Scutts SR, Gonzalez L (2019). Vaccinia virus BBK E3 ligase adaptor A55 targets importin-dependent NF-κB activation and inhibits CD8 ^+^ T-cell memory. J Virol.

[44] Alejo A, Ruiz-Argüello MB, Ho Y, Smith VP, Saraiva M (2006). A chemokine-binding domain in the tumor necrosis factor receptor from variola (smallpox) virus. Proc Natl Acad Sci U S A.

[45] Yuwen H, Cox JH, Yewdell JW, Bennink JR, Moss B (1993). Nuclear localization of a double-stranded RNA-binding protein encoded by the vaccinia virus E3L gene. Virology.

[46] Yang Z, Cao S, Martens CA, Porcella SF, Xie Z (2015). Deciphering poxvirus gene expression by RNA sequencing and ribosome profiling. J Virol.

