# Supplementary material for: Leaky scanning translation generates a second A49 protein that contributes to vaccinia virus virulence
Source: J Gen Virol. 2020 Feb 25;101(5):533–41. doi: 10.1099/jgv.0.001386 (PMC7414448; doi:10.1099/jgv.0.001386)

Supplementary Figure 1. Expression of A49 oofATG from VACV-infected cells. HEK-293T cells were infected at 5 p.f.u. / cell with WT VACV (vWT) or a mutant virus (voofATG). At 16 h p.i. cells were harvested and cell extracts were analysed by SDS-PAGE and immunoblotting with antibodies to VACV proteins A49 and D8 or α-tubulin. Red arrows indicate the positions of the large (L) or small (S) A49 proteins.

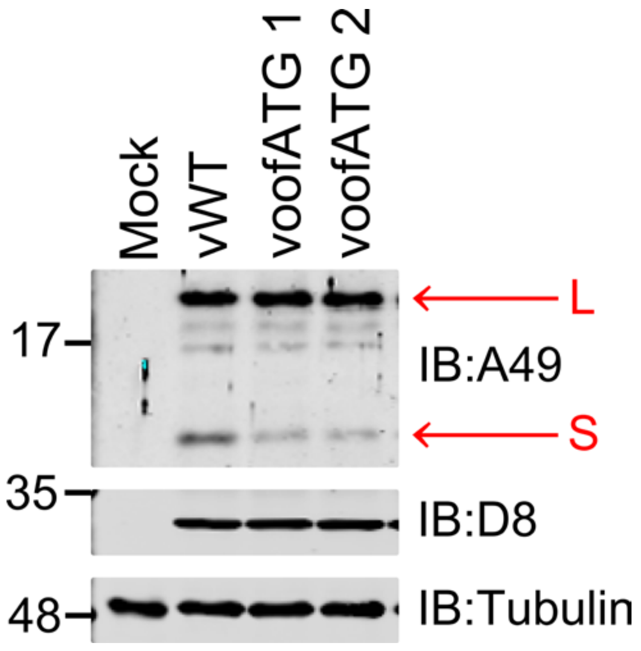

Supplement: Supplementary material 1 [file jgv-101-533-s001.pdf]
